# Damage of irradiated teeth by ultrasonic scaling: an in vitro study

**DOI:** 10.1007/s00520-026-10992-5

**Published:** 2026-07-18

**Authors:** Jana Krapež, Marko Polajnar, Klemen Leopold, Jakob Peterlin, Attila Šarvari, Mitjan Kalin, Aleš Fidler

**Affiliations:** 1 Department of Endodontics, University Medical Center Ljubljana, Ljubljana, Slovenia; 2 Department of Dental Diseases, Faculty of Medicine, University of Ljubljana, Ljubljana, Slovenia; 3 Laboratory for Tribology and Interface Nanotechnology, Faculty of Mechanical Engineering, University of Ljubljana, Ljubljana, Slovenia; 4 Department of Prosthodontics, Faculty of Medicine, University of Ljubljana, Ljubljana, Slovenia; 5 Institute for Biostatistics and Medical Informatics (IBMI), Faculty of Medicine, University of Ljubljana, Ljubljana, Slovenia; 6 Department of Radiotherapy, Institute of Oncology Ljubljana, Ljubljana, Slovenia

**Keywords:** Ultrasonic scaling, Irradiated teeth, Radiation caries, Head and neck cancer, Osteoradionecrosis

## Abstract

**Purpose:**

To test whether ultrasonic (US) scaling–induced hard-tissue surface loss on irradiated teeth depends on irradiation dose, type of scaler tip, angulation and orientation and tooth region.

**Methods:**

In vitro study on 24 human molars irradiated with 0, 30, or 70 Gy. US scaling was robot-standardized (force 1 N, tips G6 and P20, angulation 15° and 45°, orientation back and lateral) with medium power and water spray. Surface loss was graded on intraoral scans by three blinded observers on a six-band ordinal 0–5 scale (increasing with loss depth) and analyzed with cumulative-logit ordinal mixed models for median and maximum depth.

**Results:**

30 Gy significantly increased the odds of deeper surface loss with the back orientation (median: *β* = 2.43, *p* = 0.039; max: *β* = 2.10, *p* = 0.022); no 70 Gy contrast reached significance, indicating a dose-threshold effect. The G6 tip increased the odds of deeper damage substantially more than P20 across both depth outcomes (all *p* < 0.001), and 45° angulation together with the back orientation further amplified peak damage. The root was more susceptible than the crown (*β* ≈ 2.95, *p* < 0.001).

**Conclusions:**

US instrumentation of irradiated teeth produced dose- and technique-dependent hard-tissue surface loss. Slim periodontal tips (P20) used at shallow angulation (15°) with a lateral orientation were associated with minimal surface loss across all doses and positions, whereas wider tip (G6) at 45° with the back orientation produced consistent measurable wear, amplified on the root and by prior irradiation.

**Supplementary Information:**

The online version contains supplementary material available at 10.1007/s00520-026-10992-5.

## Introduction

About 30% of head and neck cancer (HNC) patients treated with radiotherapy (RT) develop radiation-related caries (RC), typically within 6 to12 months and with a predilection for cervical surfaces and incisal/cuspal tips [1–3]. RC arises via two pathways that act in parallel: indirect effects, dominated by RT-induced hyposalivation with secondary changes in diet, buffering capacity, and the oral microbiome; and direct effects, in which radiation alters the chemical composition and mechanical properties of enamel, dentine, and cementum, reducing their demineralization and wear resistance [4–7]. Clinically, RC is often rampant: Teeth cavitate rapidly, fractures are common, and the lesions can advance to pulpal involvement within weeks to months, raising the risk of osteoradionecrosis—a rare but serious complication with limited treatment options and high treatment cost. Maintenance of oral health in this population is therefore imperative [8, 9].

Multinatioanl Supportive Care in Cancer / International Society of Oral Oncology (MASCC/ISOO) provides global recommendations for HNC patients before, during, and after RT [10, 11] but does not specify post-RT follow-up intervals or procedure-level protocols for oral maintenance care. Practice patterns remain heterogeneous between regions [12, 13], and Slovenian national recommendations were issued after reviewing comparable European systems [14]. A gap remains for ultrasonic (US) instrumentation: The frequency and safe operating parameters are not standardized for HNC patients.

US scalers are routinely used for calculus removal and plaque disruption as part of professional oral maintenance care. Their tissue impact depends on multiple interacting factors—including device type, power setting, clinician technique, applied force, duration and stroke dynamics, tip type and wear, angulation, orientation, and the patient’s oral condition—making even nominally standardized scaling sessions highly variable in practice. Clinicians’ technical knowledge of US-scaler behavior is often inadequate [15, 16], and incorrect power settings, excessive force, worn tips, steep angulation, or uncontrolled stroke can damage tooth surfaces [17], resulting in iatrogenic plaque accumulation and caries progression at roughened surfaces [18]. Because US scalers are used on both crown and root regions, which consist of enamel, cementum, and dentine with markedly different mechanical and chemical properties, the same operating parameters produce different degrees of surface loss on different substrates.

Susceptibility to such damage is likely increased in HNC patients after RT. Depending on tumor location and field design, individual teeth may be exposed to cumulative doses up to 70 Gy, and RT has a dose-dependent impact on dental hard tissues [4]. RT alters chemical composition [7] and reduces micro- and nanomechanical properties [19, 20], yielding decreased wear resistance, increased surface roughness, and microcracking [6, 21]. Mechanical vulnerability of irradiated dentine has been demonstrated for endodontic instrumentation—both for increasing root-canal preparation taper [22] and across different instrumentation kinematics [23], but no study has examined how US-scaling parameters translate to irradiated enamel and cementum, and current supportive-care recommendations for HNC patients therefore lack evidence-based guidance on safe US-scaling parameters.

The aim of the study was to evaluate whether US scaling–induced hard-tissue surface loss on irradiated teeth depends on irradiation dose, scaler tip type, angulation and orientation and tooth region (crown vs root).

## Materials and methods

The study was conducted in accordance with the Declaration of Helsinki and was approved by the Commission of the Republic of Slovenia for Medical Ethics (N0120-351/2021/6). Written informed consent was obtained from all patients before tooth extraction.

### Specimen selection and preparation

From 40 extracted human molars, 24 fully erupted third molars were selected because they were readily available, comparable, and most likely to fulfill the inclusion criteria - without visible carious lesions, structural defects, or restorations were selected. Teeth were cleaned of soft tissues, stored in saline with 0.08% thymol until preparation, and sectioned longitudinally into buccal and lingual halves using a diamond-coated band saw (Minitom, Struers, Copenhagen, Denmark). Sections were mounted on acrylic plates, brushed with a nylon prophylaxis brush (BECHT4PROPHY, Alfred Becht, Offenburg, Germany), polished with prophylaxis paste (Proxyt-Fine, Ivoclar Vivadent, Schaan, Liechtenstein), and stored in phosphate-buffered saline (PBS) with 0.08% thymol. Teeth were randomly assigned to three groups (*n* = 8 each): D0 (nonirradiated), D3 (30 Gy), and D7 (70 Gy).

### Irradiation protocol

Teeth were placed in a 6-well plate (Eppendorf, Hamburg, Germany) immersed in PBS with 0.08% thymol at room temperature, with the solution replaced weekly. Irradiation followed a clinical schedule of 5 fractions/week at 2 Gy per fraction on a linear accelerator (Varian Unique Power, Varian Medical Systems, Palo Alto, CA, USA) with a 6 MV photon beam: 30 Gy over 3 weeks for D3 and 70 Gy over 7 weeks for D7. Post-irradiation, four shallow reference pits were prepared at the tooth–acrylic interface using a small round diamond bur to facilitate spatial registration of surface scans.

### Ultrasonic scaling

**Fig. 1:**
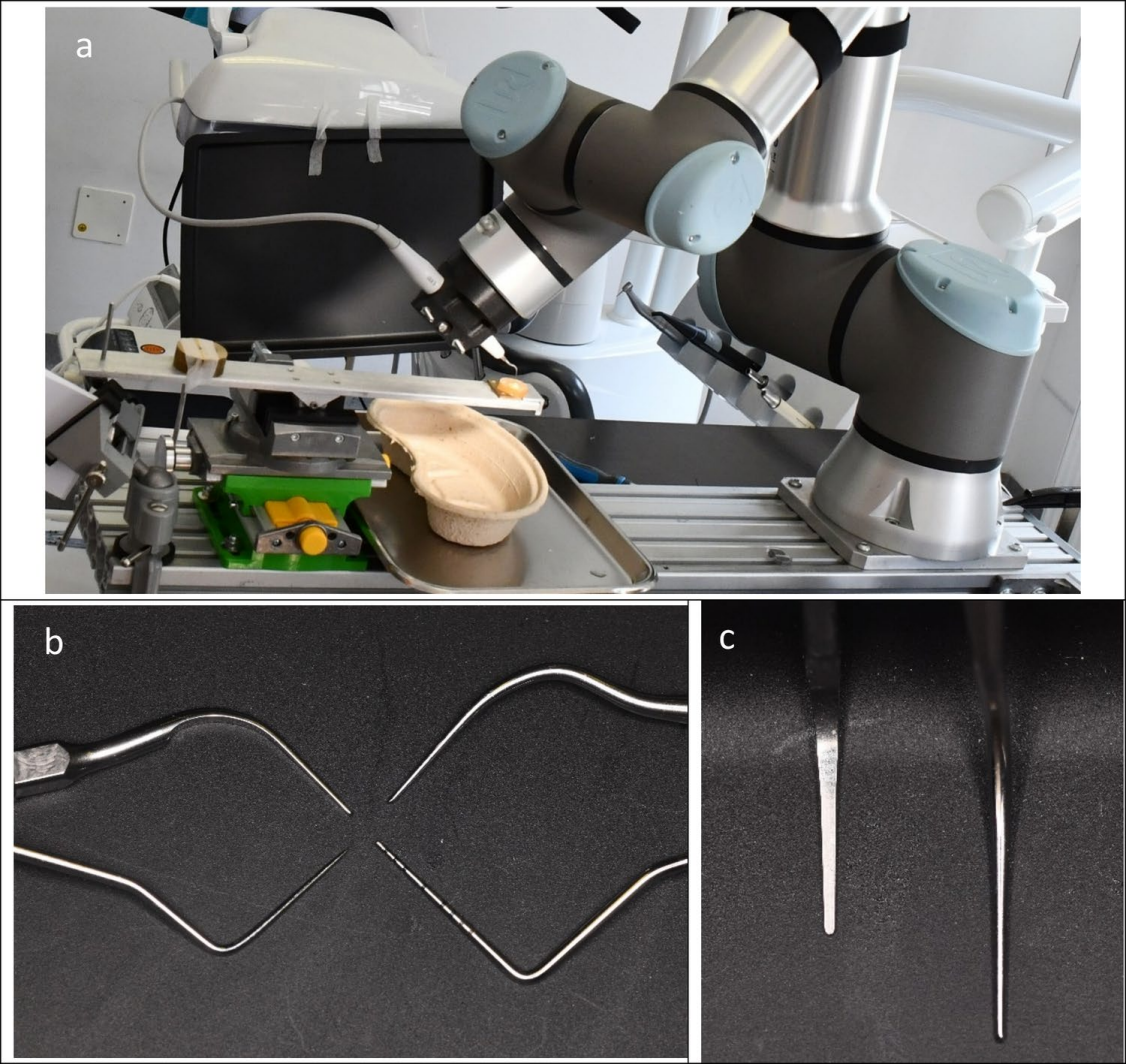
Experimental setup for robot-assisted ultrasonic (US) scaling. **a** Overview of the robotic rig, with the UR5 collaborative robot (right) holding the US handpiece above a tooth specimen mounted on a custom counterweight arm that maintains a constant 1 N contact force. **b** Close-up view of lateral orientation of the G6 tip (upper left) and P20 tip (upper right), shown together with a sharp dental explorer (lower left) and a periodontal probe (lower right) as reference objects. In this projection, the true tip relief is not fully visible. **c** Back orientation of the G6 tip (left) and P20 tip (right), in which the surface relief is more clearly seen

The study evaluated only one US model and two tip designs, so the findings may not be applicable to other US models or tip designs from different manufactures. Operator bias and motion variability were eliminated by standardizing both tip force and tip motion. Tip contact force was held at 1 N—a value corresponding to the upper end of the clinically recommended range [24]—using a custom metal rig [25] in which the tooth was fixed at one end of a pivoted arm and a counterweight at the other, ensuring a constant contact force irrespective of tip trajectory (Fig. 1a). Tip position, orientation, and angulation were standardized with a collaborative robot (UR5, Universal Robots, Odense, Denmark) programmed to execute linear sweeps with submillimeter repeatability. Each specimen was subjected to a standardized scaling pattern of four longitudinal lines 5 mm in length, spaced 0.75 mm apart, executed at a linear speed of 2.5 mm·s⁻^1^ for 10 s per line. The four lines on a given tooth were each allocated to one fixed combination of tip orientation (back or lateral) and tip angulation (15° or 45°): L15 (lateral, 15°), L45 (lateral, 45°), B45 (back, 45°), and B15 (back, 15°), as illustrated in Fig. 1b–e. This full within-tooth crossing of the two technique parameters allowed each tooth to serve as its own control across orientation × angulation conditions, removing between-tooth variability from these contrasts.

US scaling was performed with a Varios 970 LUX piezoelectric scaler (NSK Nakanishi, Tochigi, Japan) set to medium power with water cooling; water flow was verified at 18 mL/min before each session. Within each dose group, teeth were randomly subdivided into two subgroups (*n* = 4 each) instrumented with either a G6 tip (General tip 6, wider cross-section, intended for supragingival calculus removal) or a P20 tip (Periodontal tip 20, slim periodontal design, intended for subgingival debridement). Tip integrity was verified before each session, and tips were replaced after every eight teeth to control for wear-related drift in vibration behavior.

### Surface evaluation

**Fig. 2:**
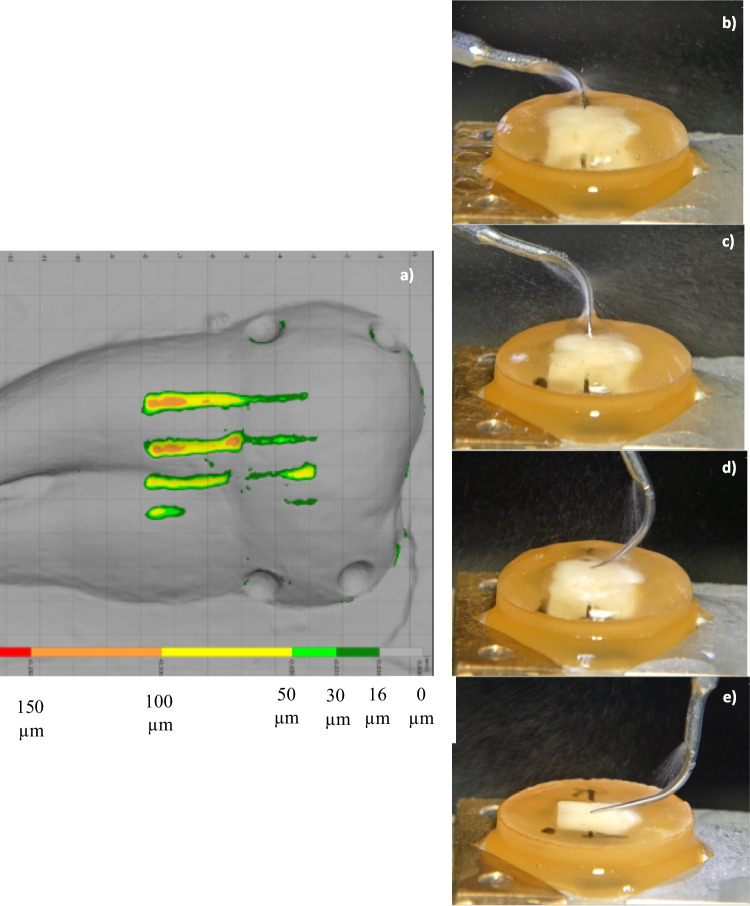
**a** Color-coded deviation scale used to grade ultrasonic-scaling damage. Perpendicular distances between the baseline and post-instrumentation meshes were computed at corresponding mesh points and binned into six depth bands, each corresponding to an ordinal damage category: category 0 (< 16 µm, gray, not visualized), category 1 (16–33 µm, light green), category 2 (33–50 µm, dark green), category 3 (50–100 µm, yellow), category 4 (100–150 µm, orange), and category 5 (≥ 150 µm, red). The image shown is a representative specimen (D0-3; 0 Gy, G6 tip). **b–e** Close-up views of the four G6 tip orientation and angulation combination applied to the four longitudinal lines on each tooth: **b** lateral orientation at 15° angulation (L15); **c** lateral orientation at 45° angulation (L45); **d** back orientation at 45° angulation (B45); **e** back orientation at 15° angulation (B15)

The pre-defined 0-5 scale was derived from the color-coded depth intervals of the intraoral scanning (IOS) software. These tresholds were not previously externaly validated for irradiated dental hard tissues and were therefore used as a study-specific ordinal grading system. Reproductibility of the grading was assessed by blinding observers using inter- and intra.observer reliability analysis. Hard-tissue loss was measured with an intraoral scanner (TRIOS 4, 3Shape, Copenhagen, Denmark) before (baseline) and after instrumentation (post-scaling). All scans were performed under standardized dimmed light conditions, with a controlled working distance, and all surfaces were gently dried with a dental airstream blower immediately before scanning. Raw scan data were exported as Standard Tesselation Language (STL) meshes and coded by an independent operator to ensure blinded downstream evaluation. STL files were imported into GOM Inspect metrology software (Carl Zeiss Gessellschaft fuer Optische Messetechnick - GOM, Metrology, Braunschweig, Germany) and processed in two registration steps: first, a global best-fit registration aligned baseline and post-scaling meshes; then, a local best-fit registration was applied while explicitly excluding the four instrumented lanes, using the reference pits and undisturbed surrounding regions as registration anchors. Surface loss was then quantified by a surface-comparison algorithm that computes the perpendicular distance between corresponding points on the two meshes and visualized as a color-coded deviation map with six discrete colors centered on prespecified depth thresholds: gray < 16 µm (category 0), light green 16–33 µm (1), dark green 33–50 µm (2), yellow 50–100 µm (3), orange 100–150 µm (4), and red ≥ 150 µm (5) (Fig. 2). For each of the four lines on each specimen, the crown and root areas were graded separately by three blinded observers, who recorded two outcomes per area: the predominant depth category, defined as the color covering the largest proportion of the line (referred to as median), and the maximum depth category observed anywhere along the line (referred to as max). Two observers repeated all assessments after 1 week, enabling intrarater reliability analysis. In cases of disagreement, the most experienced observer (A.F., > 10 years of intraoral-scan evaluation experience) adjudicated the final rating. Inter- and intraobserver reliability was summarized with quadratic-weighted *κ* (with bootstrap 95% confidence intervals) and percent agreement.

Representative specimens were additionally characterized with digital microscopy (DM) and green-light interferometry (GLI). DM was performed with a 3D digital microscope (Hirox HRX-01, Limonest, France) at 20 ×, 50 ×, and 120 × (5 µm lateral resolution); 20 × was used to show the whole tooth with the four longitudinal lines and 50 × and 120 × to show crown and root detail. GLI was performed with a 3D optical interferometer (ContourGT-K0, Bruker, Billerica, MA, USA) using a 5 × lens with a field of view of 0.55 ×, giving a scan area of 1.73 × 1.30 mm and a lateral resolution of 1.346 µm; images were acquired at the crown, root, and CEJ regions of each of the four lines.

### Statistical analysis

We analyzed the data using R (version 4.2.3). We fitted ordinal mixed models using the ordinal package (version 2023.12-4) [26] and performed multiple comparisons using single-step procedures from the multcomp package (version 1.4.25) [27]. The statistical significance level was set at 0.05. We have used the following ordinal mixed model with cumulative logit link to model our data:

$$y\sim 1+P+T*A+D*O+\left(1|ID\right)$$

In the above formula, *y* indicates the rating, *P* position (crown or root), *T* tip (G6 or P20), *D* dose (0, 30, or 70 Gy), *A* angulation (15° or 45°), and *O* orientation (back or lateral) and the term $\left(1|ID\right)$ indicates tooth-specific random intercept term that accounts for between-tooth variability and the correlation among repeated measurements taken within the same tooth where *ID* stands for the ID of a tooth. The operator ∗ indicates the presence of both terms as well as the presence of the interaction term. Analysis was performed for the median and maximum depths separately.

## Results

Twenty-four teeth were instrumented; two were excluded from the depth analysis because surface complexity caused irregular robot motion (final *n* = 22). Ordinal grading showed excellent intra- and interobserver reliability (quadratic-weighted *κ* = 0.97–0.99; percent agreement 88–96%) and excellent agreement with the final consensus ratings (*κ* = 0.98–0.99; percent agreement ≈ 95%).

Ordinal mixed-effects models were fitted for median and maximum damage depths (Table 1). The random intercept for individual teeth accounted for 28.8% of the total variance for median depths and 13.3% for maximum depths (ICC = 0.288 and 0.133, respectively), justifying the use of mixed-effects modelling to account for biological differences between specimens.

**Table 1:** Ordinal mixed-model estimates for median and maximum damage depths. Estimates (*β*) represent log-odds; positive values indicate higher odds of deeper damage. *p* values and 95% confidence intervals are multiplicity-adjusted. Rows under dose and tip × angle represent simple effects—the combined main and interaction effects evaluated at the specified level of the interacting factor—rather than standalone interaction coefficients. ^*^p < 0.05

Variable	Contrast	Median depth: *β* [95% CI]	*p*	Maximum depth: *β* [95% CI]	*p*
Dose (30 Gy)	Back vs. 0 Gy	2.43 [0.07, 4.78]	0.039^*^	2.10 [0.20, 4.01]	0.022^*^
	Lateral vs. 0 Gy	0.62 [− 1.47, 2.71]	0.965	0.96 [− 0.85, 2.77]	0.639
Dose (70 Gy)	Back vs. 0 Gy	1.97 [− 0.47, 4.41]	0.180	1.06 [− 0.88, 3.01]	0.606
	Lateral vs. 0 Gy	− 0.25 [− 2.48, 1.98]	1.000	− 0.49 [− 2.37, 1.39]	0.982
Tip × angle	G6 at 15° vs. P20	7.16 [4.18, 10.14]	< 0.001^*^	5.46 [3.51, 7.42]	< 0.001^*^
	G6 at 45° vs. P20	7.04 [4.56, 9.52]	< 0.001^*^	8.35 [5.92, 10.79]	< 0.001^*^
Position	Root vs. crown	2.95 [1.73, 4.18]	< 0.001^*^	2.93 [1.89, 3.96]	< 0.001^*^

### Radiation dose and tip orientation

The dose effect on damage severity was orientation-dependent (dose × orientation interaction). With the back orientation, 30 Gy significantly increased the probability of deeper damage compared with non-irradiated controls (median: *β* = 2.43, *p* = 0.039; max: *β* = 2.10, *p* = 0.022). The 70 Gy × back contrast did not reach significance in either model, and no lateral-orientation contrast reached significance at any dose.

### Ultrasonic tip and angulation

Tip type and angulation strongly influenced both median and maximum damage. For median depth, G6 was more aggressive than P20 with nearly identical estimates at 15° (*β* = 7.16) and 45° (*β* = 7.04). For maximum depth, G6 was again more aggressive at 15° (*β* = 5.46), and increasing the angulation to 45° further exacerbated peak damage (*β* = 8.35). The tip × angle interaction reached significance in the maximum-depth model but not the median-depth model, indicating that the combined effect of tip type and angulation manifests more consistently as peak damage than as a shift in the predominant damage pattern.

### Crown versus root

Anatomical position was a highly significant predictor. The root was more susceptible than the crown across all conditions in both the median-depth (*β* = 2.95) and maximum-depth (*β* = 2.93) models (both *p* < 0.001).

### Marginal predicted probabilities (Fig. 3)

**Fig. 3:**
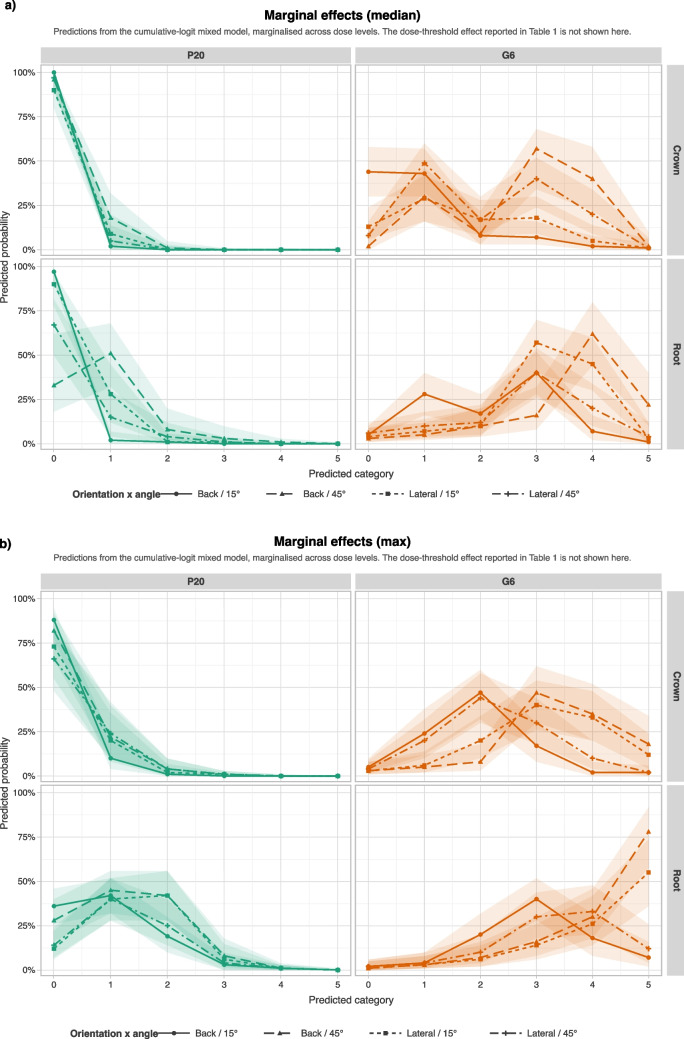
Marginal predicted probabilities. **a** Median-depth summary. **b** Maximum-depth summary. Of each ordinal damage category (0 = no loss; 5 = deepest loss, as defined in Fig. 2) obtained from the cumulative-logit ordinal mixed model (Table 1) and marginalized across radiation dose (0, 30, and 70 Gy). Rows show anatomical position (crown, root) and columns show scaler tip (P20, green; G6, orange). Within each panel, the four curves represent the orientation × angulation combinations of the ultrasonic tip (back/15°, back/45°, lateral/15°, lateral/45°). Shaded ribbons are unadjusted 95% confidence intervals. The dose-threshold effect (significant increase from 0 to 30 Gy with the back orientation; no further increase at 70 Gy) is reported in Table 1 and is not depicted here

Marginal predicted probabilities of each damage category, marginalized across dose, are shown in Fig. 3 as a function of tip (columns), anatomical position (rows), and orientation × angulation (four curves within each panel); median (Fig. 3a) and maximum (Fig. 3b) summaries are presented separately. P20 concentrated the predicted probability near category 0 on the crown and shifted only modestly toward categories 1–2 on the root, with the four orientation × angulation curves tightly clustered in both positions. G6 shifted the predicted peak into categories 3–4 on the crown and 4–5 on the root, and the four curves fanned out with widening confidence ribbons, most markedly on the root, with back/45° carrying the most probability mass in the deep-damage categories.

### Qualitative surface assessment

Four representative specimens—D0-3 (0 Gy, G6), D3-1 (30 Gy, G6), D0-6 (0 Gy, P20), and D3-8 (30 Gy, P20)—were examined with IOS, DM, and GLI to corroborate the patterns identified in Table 1 and Fig. 3. Per-specimen descriptions and all numerical depth values are reported in Supplement 1 (Figures S1–S8). Across all three modalities, the dominant tip × position pattern was confirmed: G6 specimens showed clearly delineated tracks in all four lanes, wider and deeper on the root than on the crown, with the deepest track observed in the irradiated specimen (D3-1); P20 specimens showed only minimal tracks at low DM magnification, scattered light-green IOS flecks, and GLI Y-profiles with no measurable wear in either region of D3-8. GLI X-profiles on G6 roots displayed pronounced short-range peaks and valleys, whereas P20 tracks were uniform on both crown and root. Two qualitative observations are not captured by the statistical model: At the cemento-enamel junction (CEJ) the abrupt change in surface relief and tissue properties frequently induced tip skipping and discontinuity of the groove, most conspicuous in the G6 specimens and largely absent with P20; and localized craters were frequently observed at both track endpoints.

## Discussion

In this in vitro model, iatrogenic surface loss during US scaling of irradiated teeth was a predictable outcome of tip geometry, anatomical position, radiation dose, and—to a lesser extent—tip angulation and orientation. Tip type and position emerged as the two dominant determinants of damage severity, with angulation and orientation acting as modifiers whose influence grew as substrate vulnerability increased. To the best of our knowledge, this is the first parametric study to quantify US-scaling damage on irradiated human teeth with full within-tooth control over tip orientation and angulation, graded on an ordinal scale that preserves the clinical relevance of the color-coded deviation map, and analyzed with a mixed model that accounts for tooth-level clustering. All three experimental factors (dose, tip, tooth region) significantly affected surface damage, consistent with evidence that RT alters dental hard tissues, reducing their hardness and wear resistance and increasing their susceptibility to mechanical removal [4–7, 19–21].

The consistently higher damage scores observed on root surfaces across all groups reflect the inherent vulnerability of cementum and dentine. Cementum is thin, typically 50–200 µm at the cervical third [28] and once perforated exposes dentine, whose tubular architecture amplifies wear once a track is initiated. This anatomical vulnerability coincides with the cervical predilection of RC [1, 2], making the root surface both the site most exposed to caries and the site least tolerant of instrumentation error. Because enamel, dentine, and cementum do not regenerate, even small hard-tissue losses are clinically meaningful [4, 28, 29]; consistent with this, earlier in vitro data suggest that US scaling removes less root substance than hand instrumentation at higher forces [30–32], supporting a minimal-removal strategy in these patients.

Mechanistically, the pattern reflects tissue properties. Cementum and dentine are softer and less wear-resistant than enamel, and RT amplifies this gradient by making tissues more brittle. A slim tip vibrating laterally delivers more of a a shaving motion that brittle irradiated tissue tolerates, whereas a wider tip at 45° maybe delivers more hammer-like motion. This is most apparent on the root: On crown surfaces, P20 concentrated nearly all predicted probability at the lowest damage level regardless of technique, suggesting tip geometry alone is sufficient to protect enamel under the tested conditions and that angulation and orientation become relevant only as substrate vulnerability increases. The protective behavior of P20 was preserved even at 45° angulation (median: *β* = 7.16), whereas G6 produced consistent wear across all tested conditions. Because biofilm disruption rather than hard-deposit removal is the goal in irradiated patients, a less aggressive technique appears clinically sufficient. Consistent with this, the tip × angle interaction reached significance only for maximum depth, indicating that the combined effect of tip geometry and angulation manifests mainly as localized peak damage—worst-case damage at turning points and anatomical transitions—rather than as a shift in the predominant damage pattern. The qualitative observation of tip skipping and groove discontinuity at the cemento-enamel junction provides a mechanical rationale: The abrupt change in relief and tissue properties at the CEJ forces the tip out of steady-state contact, producing short-range impulsive loading that the maximum-depth outcome captures but the modal category does not.

The dose effect followed a threshold rather than a linear pattern. 30 Gy significantly increased damage with the back orientation (median: *β* = 2.43, *p* = 0.039; max: *β* = 2.10, *p* = 0.022), whereas no 70 Gy contrast and no lateral-orientation contrast reached significance in either model. The nonsignificant trend at 70 Gy likely reflects a plateau in tissue alterations or increased specimen heterogeneity at higher doses, consistent with reports that the radiation-induced loss of organic matrix and alteration of hydroxyapatite occur early and saturate before the maximum clinical dose is reached [6, 7, 19]. A similar vulnerability of irradiated dentine has been reported beyond surface scaling: Pauletto et al. showed that mesial roots exposed to 60 Gy exhibited significantly reduced biomechanical performance with increasing preparation taper [22], and Bertolini et al. extended this finding to different endodontic instrumentation kinematics [23]. Both studies used a single fixed radiation dose and could not resolve whether the effect is dose-dependent; the present data suggest that much of the mechanical vulnerability is already established at 30 Gy, consistent with an early-saturating threshold across mechanical interventions on irradiated teeth [33, 34]. This threshold behavior has a practical implication: Because the dose delivered to an individual tooth depends on its position relative to the target volume and on the RT planning parameters, the local dose is rarely known at the chairside, and any attempt to adjust technique based on an estimated tooth-level dose will be limited by this uncertainty. A more conservative rule—treat any tooth in an irradiated field with the same caution one would apply to a tooth at 30 Gy or above—is both safer for the patient and easier to implement in routine practice.

The limitations of this study, including a sample size (*n* = 24) that limits precision for subgroup analyses, must be considered alongside measurement challenges inherent to natural teeth. Of the 24 teeth enrolled, two were excluded because surface complexity caused irregular robot motion (final *n* = 22), further limiting precision for subgroup contrasts. Natural teeth best approximate the clinical substrate, but their relief-rich topography made precise profilometric and tribological assessment technically demanding, as 3D optical and stylus profilometry could not reliably separate raster traces from intrinsic surface features [35, 36]. Complex tooth morphology occasionally led to incomplete grooves or increased depth at turning points, and localized craters were observed at both track endpoints: Both reflect robot deceleration and reversal at sweep ends rather than steady-state instrumentation and should be interpreted as artefacts of the standardized motion protocol rather than clinically representative damage patterns. Beyond specimen-level constraints, the in vitro design has intrinsic limitations: Saliva, acquired pellicle, and bacterial biofilm—which modulate US energy transfer and tissue response in vivo—were absent; one US device and one pair of tip designs were tested, so extrapolation to other devices and tips should be cautious; and irradiation was delivered to extracted teeth in PBS rather than in a living jaw, excluding vascular and pulpal responses that modify tissue behavior in patients. Prospective clinical studies are needed to validate these observations under realistic biological conditions.

Within these constraints, the findings provide a mechanical framework for clinical evaluation. Under the tested conditions, slim periodontal tips (e.g., P20) at shallow angulation (15°) with lateral orientation produced minimal surface loss across all doses and both anatomical positions, whereas wider tips at 45° with back orientation produced consistent wear, amplified on the root and by prior irradiation. Light contact force, moderate power, stable water flow, and short intermittent strokes—parameters used in this study—offer a plausible starting point for clinical protocols aimed at biofilm disruption rather than hard-deposit removal in irradiated patients, pending prospective validation. These findings have two clinically relevant implications: Tip wear alters both tip geometry and resonance behavior, so regular replacement of P20 tips is essential—a worn slim tip can approach the aggressiveness of a new general-purpose tip; and because irradiated patients rely heavily on their oral-hygiene behavior between recall appointments, the same minimal-removal principle should guide at-home-care counseling, including soft brushes, fluoride-rich regimens, and early clinical review of cervical surface changes. Specific areas for future research are there for many - evaluating different US systems, power settings, tip designs, and long term clinical studies outcomes in irradiated patients.

## Supplementary Information

Below is the link to the electronic supplementary material.

ESM 1 (DOCX 11.9 MB)

## Data Availability

No datasets were generated or analyzed during the current study.
