# Supplementary material for: Damage of irradiated teeth by ultrasonic scaling: an in vitro study
Source: Support Care Cancer. 2026 Jul 18;34(8):777. doi: 10.1007/s00520-026-10992-5 (PMC13380595; doi:10.1007/s00520-026-10992-5)
Supplement: Supplementary file 1 — (DOCX 11.9 MB) [file 520_2026_10992_MOESM1_ESM.docx]

**Qualitative evaluation of surface changes**

The representative specimens D0-3, D3-1, D0-6 and D3-8 were evaluated by intra-oral scanner (IOS) derived colour-coded damage maps, digital microscopy (DM) at 20×, 50×, and 120× magnification and by green-light interferometry (GLI) with extracted X (transverse) and Y (longitudinal) profiles. IOS colour-coded damage maps for each specimen are shown in Figures S1, S3, S5, and S7; corresponding DM and GLI panels are shown in Figures S2, S4, S6, and S8. For G6 specimens (D0-3, D3-1), the focus was on lines 2 (lateral, 45°) and 3 (back, 45°) based on the G6 prediction pattern in Figure 3. For P20 specimens (D0-6, D3-8), the focus was on lines 1 (lateral, 15°) and 4 (back, 15°).

**D0-3 (0 Gy, G6 tip)**

The IOS colour-coded deviation map (Figure S1) shows clearly delineated tracks in all four lanes, with predominantly yellow cores (50–100 µm, category 3) and orange centres (100–150 µm, category 4) in lines 1, 2, and 3; line 4 appears narrower and discontinuous. The damage is markedly concentrated toward the root end of each lane, with the crown end showing thinner, green-edged tracks, giving a clear root-over-crown gradient consistent with the predicted pattern for the G6 tip.

All four instrumentation lines were clearly visible at every DM magnification (Figure S2a). At 50× and 120× the widths of lines 1 and 2 were greater on the root than on the crown, whereas lines 3 and 4 showed no visible width difference between crown and root. For line 2, the GLI X profile on the crown was uniform with no short-range peaks or valleys, while on the root the X profile was markedly non-uniform and the 3D GLI image showed deep localised hollows within the track (Figure S2b). The Y profile for line 2 showed a track depth of approximately 50 µm on the crown and 80 µm on the root. For line 3, differences between crown and root were less apparent: the X profile showed mildly more non-uniformity on the root, and the Y profile showed comparable depths of approximately 50 µm on both crown and root (Figure S2c).


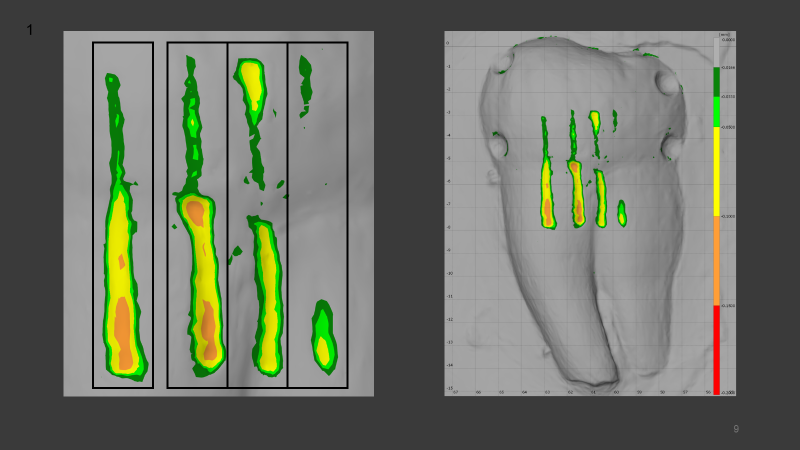


Figure S1. Intraoral scan of the D0-3 specimen (0 Gy, G6 tip) with colour-coded deviation maps: cropped close-up showing the four instrumented lanes (left) and whole-tooth view (right). Colour bands correspond to the six-step ordinal depth scale defined in Figure 2 of the main manuscript.


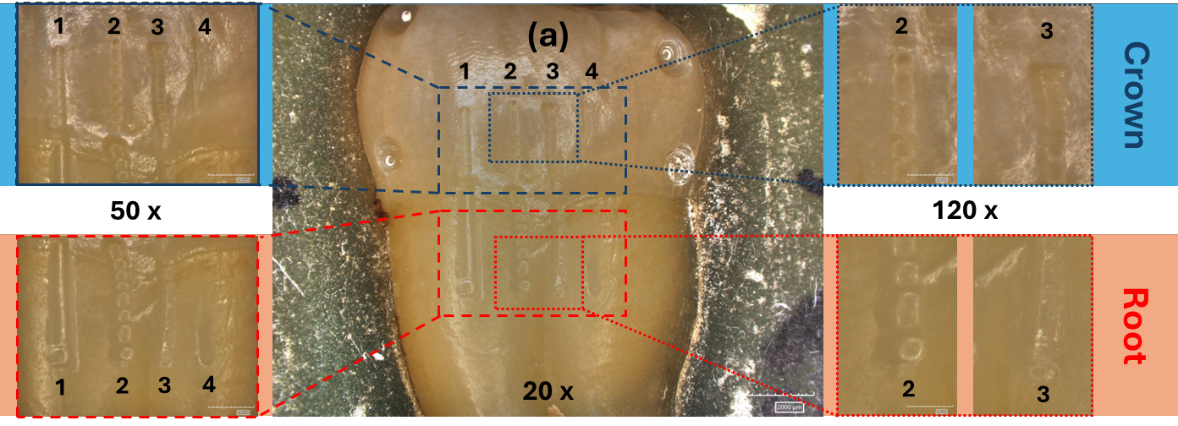

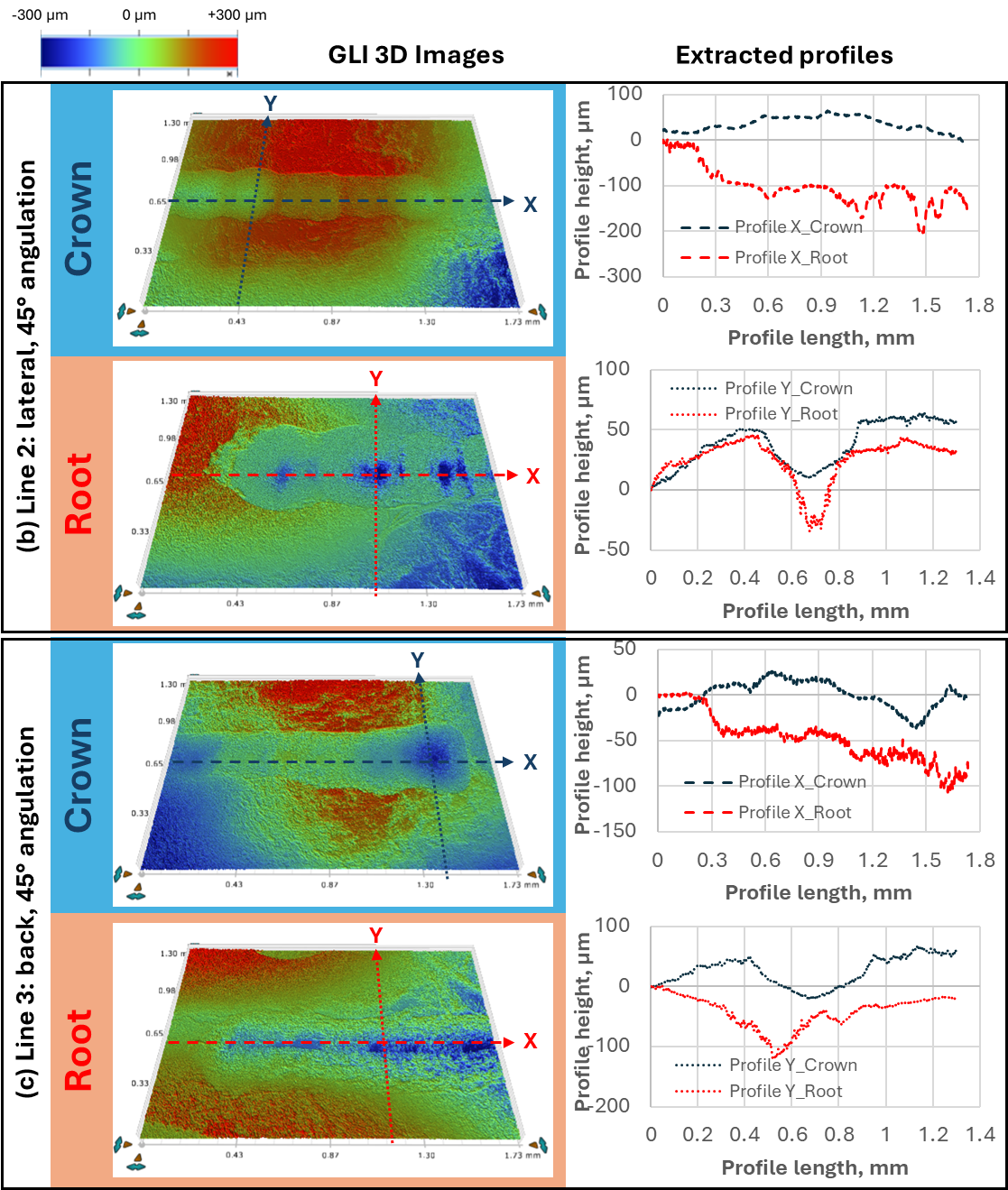


**Figure S2:** D0-3 tooth treated with 0 Gy dose and G6 tip recorded with (a) Digital Microscope at three different magnifications (20x, 50x and 120x) and with Green-light interferometry with extracted X and Y profiles for: (b) 2nd line (lateral position of tip with 45° angulation) and (c) 3rd line (back position of tip with 45° angulation).

**D3-1 (30 Gy, G6 tip)**

the IOS colour-coded deviation map (Figure S3) shows more extensive damage than the non-irradiated G6 specimen (S1). All four lanes exhibit wide yellow cores with large orange centres, and line 3 additionally shows isolated red patches (≥150 µm, category 5) at the root end — the only occurrence of category-5 damage in the four representative specimens. The root-over-crown gradient is preserved and visually more pronounced than in D0-3, in line with the significant 30 Gy × Back effect reported in Table 1.

As in D0-3, all four lines were clearly visible at every DM magnification (Figure S4a), and lines 1 and 2 were wider on the root than on the crown while lines 3 and 4 showed no visible width difference. For line 2, the GLI X profile was more uniform on the crown than on the root, and the Y profile showed a track depth of approximately 50 µm on the crown and 120 µm on the root (Figure S4b) — the deepest track observed in any of the four specimens. For line 3, differences between crown and root were again less apparent: the X profile was mildly more non-uniform on the root, while the Y profile showed comparable depths of approximately 50 µm on both crown and root (Figure S4c).


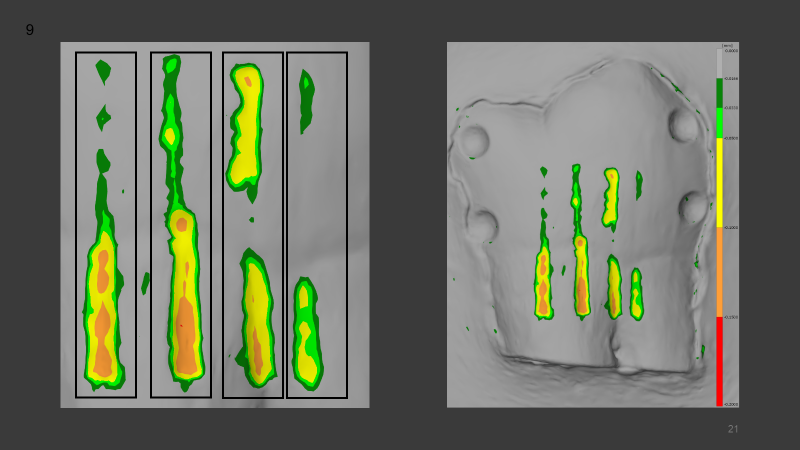


Figure S3. Intraoral scan of the D3-1 specimen (30 Gy, G6 tip) with colour-coded deviation maps: cropped close-up showing the four instrumented lanes (left) and whole-tooth view (right). Colour bands correspond to the six-step ordinal depth scale defined in Figure 2 of the main manuscript.

**
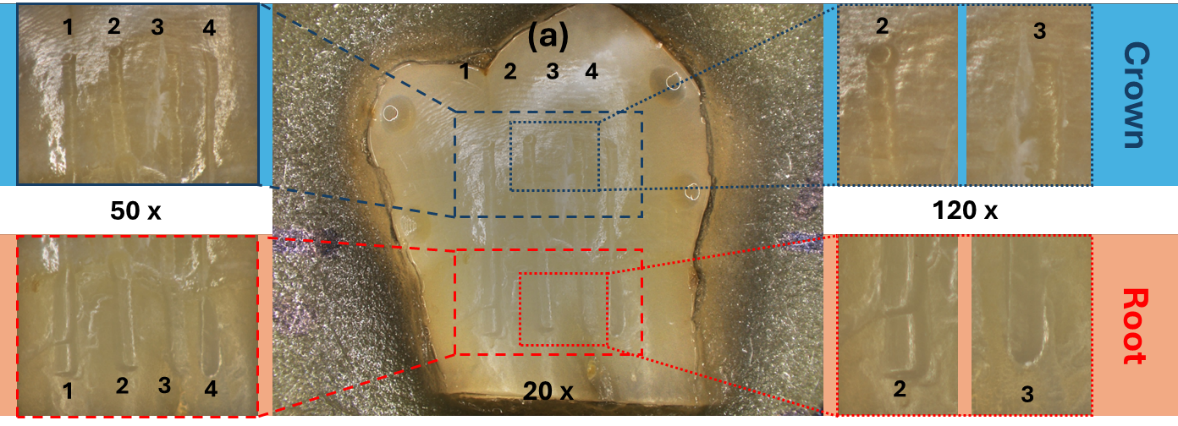
**

**
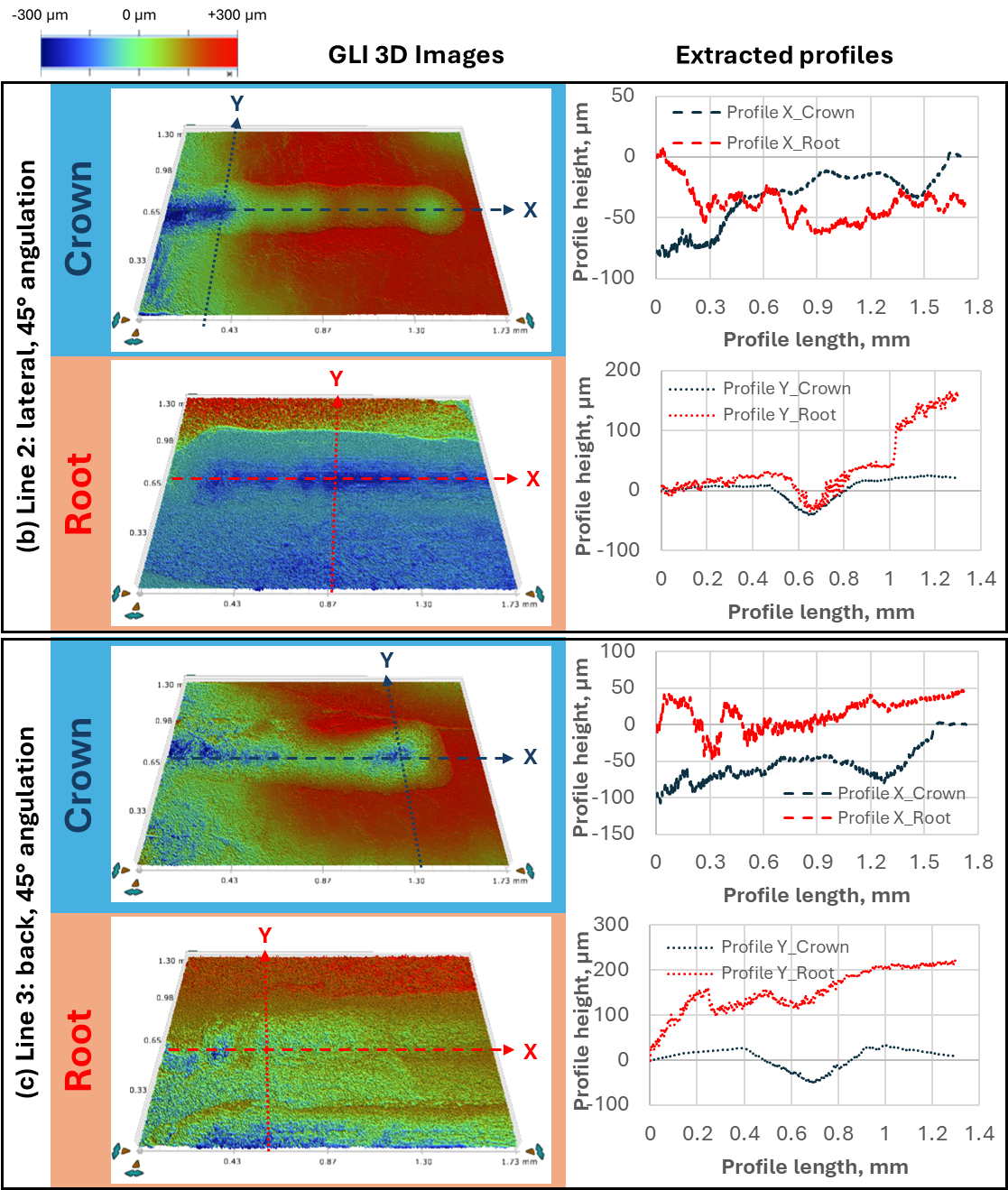
**

**Figure S4:** D3-1 tooth treated with 30 Gy dose and G6 tip recorded with (a) Digital Microscope at three different magnifications (20x, 50x and 120x) and with Green-light interferometry with extracted X and Y profiles for: (b) 2nd line (lateral position of tip with 45° angulation) and (c) 3rd line (back position of tip with 45° angulation).

**D0-6 (0 Gy, P20 tip)**

The IOS colour-coded deviation map (Figure S5) shows essentially no instrumentation-induced damage within the four lanes: only a few isolated light-green flecks (16–33 µm, category 1) are visible, while the remainder of the scanned surface remains grey (<16 µm, category 0). No yellow, orange, or red bands are present, and the whole-tooth view does not reveal discernible tracks — an IOS-level corroboration that the P20 tip at shallow angulation produces no measurable damage on non-irradiated enamel or cementum.

Instrumentation lines were barely visible at 20× DM magnification and became clearer only at 50× and 120× (Figure S6a). At 50×, lines 1 and 2 were wider on the root than on the crown, while the width difference for lines 3 and 4 was less obvious. For line 1, the GLI X profile was uniform on the crown and less uniform on the root, and the Y profile showed no detectable wear on the crown and an apparent track of approximately 55 µm on the root; this root feature may partly reflect the intrinsic tooth topography rather than instrumentation-induced wear (Figure S6b). For line 4, the X profile was uniform on both crown and root with no short-range peaks or valleys, and the Y profile showed a shallow, uniform track of approximately 15 µm on both regions (Figure S6c).


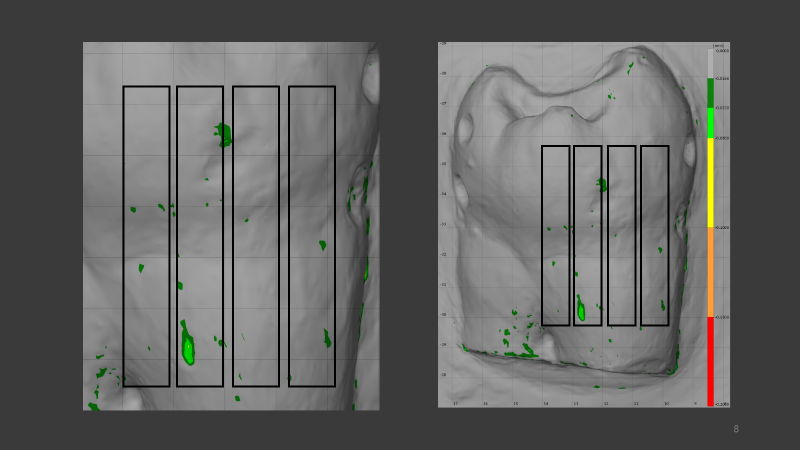


**Figure S5**. Intraoral scan of the D0-6 specimen (0 Gy, P20 tip) with colour-coded deviation maps: cropped close-up showing the four instrumented lanes (left) and whole-tooth view (right). Colour bands correspond to the six-step ordinal depth scale defined in Figure 2 of the main manuscript.

**
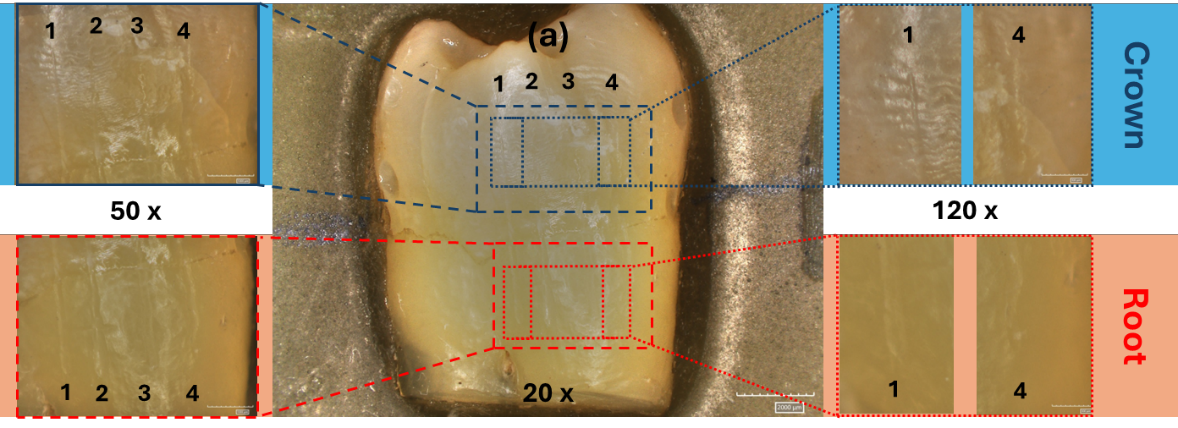
**

**
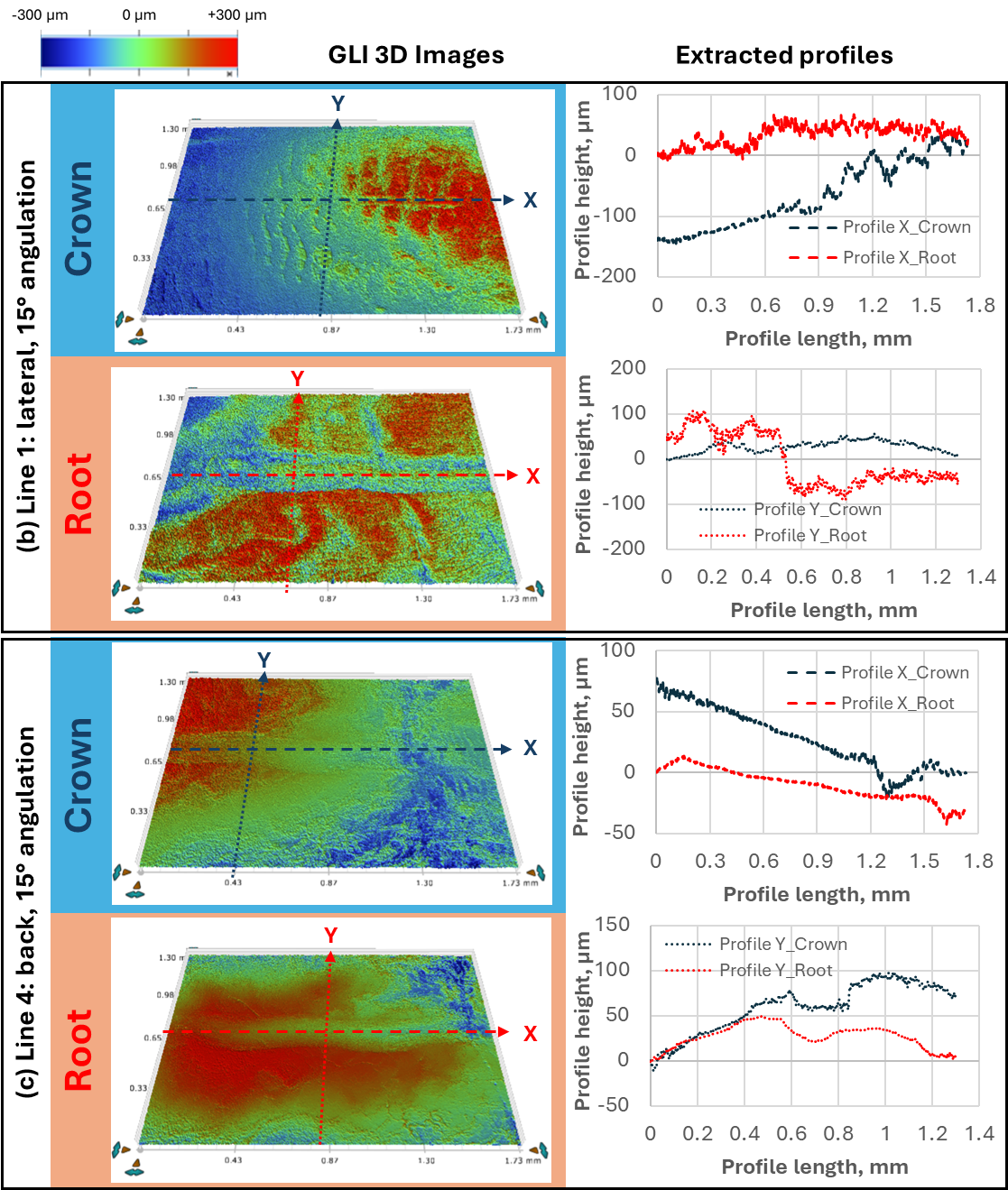
**

**Figure S6:** D0-6 tooth treated with 0 dose and P20 tip recorded with (a) Digital Microscope at three different magnifications (20x, 50x and 120x) and with Green-light interferometry with extracted X and Y profiles for: (b) 1st line (lateral position of tip with 15° angulation) and (c) 4th line (back position of tip with 15° angulation).

**D3-8 (30 Gy, P20 tip)**

The IOS colour-coded deviation map (Figure S7) shows minimal damage within the four lanes even after 30 Gy irradiation. Only a few small green patches (16–33 µm, category 1) appear at the cervical/root end of the lanes, and no yellow, orange, or red bands are present. The whole-tooth view does not reveal discernible instrumentation tracks. The similarity to the non-irradiated P20 specimen (S5) illustrates that irradiation does not appreciably worsen P20 damage at 15° angulation.

All four lines were visible at every DM magnification, but none showed a significant width difference between crown and root at 50× (Figure S8a). The 120× DM views and the 3D GLI images confirmed the absence of width differences for lines 1 and 4. For line 1, the GLI X profile was more uniform on the crown than on the root, while the Y profile showed no significant wear in either region (Figure S8b). For line 4, the X profile was similarly uniform on crown and root, and the Y profile again showed no significant wear in either region (Figure S8c).


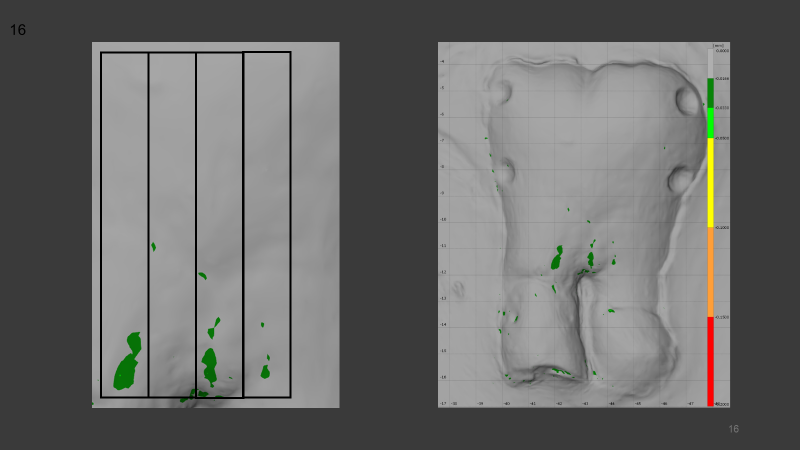


Figure S7. Intraoral scan of the D3-8 specimen (30 Gy, P20 tip) with colour-coded deviation maps: cropped close-up showing the four instrumented lanes (left) and whole-tooth view (right). Colour bands correspond to the six-step ordinal depth scale defined in Figure 2 of the main manuscript.


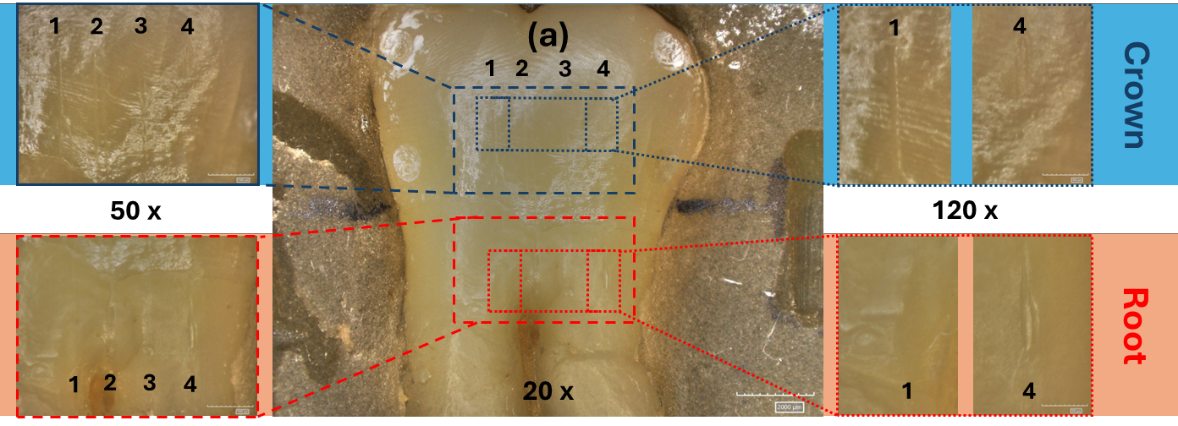
**
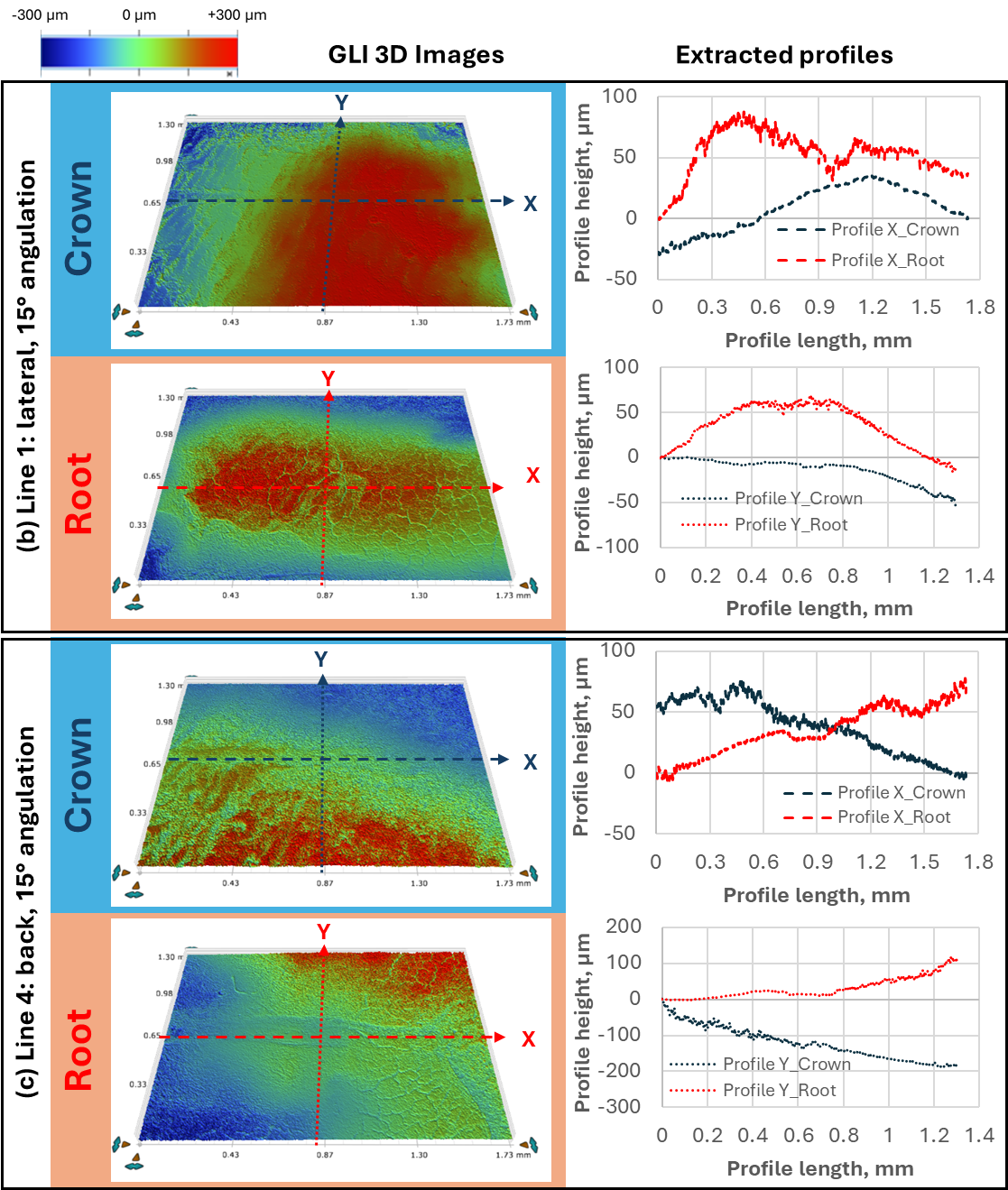
**

**Figure S8:** D3-8 tooth treated with 30 Gy dose and P20 tip recorded with (a) Digital Microscope at three different magnifications (20x, 50x and 120x) and with Green-light interferometry with extracted X and Y profiles for: (b) 1st line (lateral position of tip with 15° angulation) and (c) 4th line (back position of tip with 15° angulation).
